# 

**Published:** 1817-06

**Authors:** 


### CORRESPONDENCE.

Dr. Balfour's letters have been received. We do not find, upon a careful reperusal of the article objected to, any thing to justify his excessive warmth on the occasion. The intention of the Author appears to us to have been to invalidate the inferences drawn by the Doctor, not to accuse him of a *wilful mis-statement* of facts. Had Dr. Balfour's reply been divested of its unnecessary invective and abuse, and solely confined to elucidation of doubtful points, or a further support of his inferences by additional facts, it would have met with ready insertion. We trust the Doctor will see the propriety of our steadily rejecting all such controversial papers as can only lead to personal rancour, without in the smallest degree benefiting medical science.

Dr. Denmark's Case of Phlegmatia Dolens, with a coloured engraving, will appear in our next Number.

Mr. Mortimer's Report to Dr. Dickson will be inserted shortly.

Mr. Salt on Morbid Poisons, and the Translation of Majendie's Physiology are under Review, and will appear in rotation.

The Title and Index to this Volume will be given in our next publication.

☞ Authors and Publishers are requested to send Copies of new Works for Review as early as possible after their publication. They will be reviewed early in proportion.

### INDEX.

A

ABDOMINAL disease, curious case of 167
Abdomen, cases of wounds of the 380
Abscess of the lungs, case of 254
Acetic acid, on the use of in consumption 327
Acid, sulphuric, an ounce of, swallowed 480
Adams, Dr. on epilepsy 473
——— cases of periodical sickness 479
——— case of swallowing sulphuric acid 480
Æther, sulphuric, mode of preserving 264
——— a cure for poisoning by mushrooms 264
Albers, Dr. on a change of colour in the skin, from internal use of nitrate of silver 137
Amputation successful in the eighth month of pregnancy 264
Analysis, chemical, of the spa waters 19
Aneurism of the femoral artery, case of 125
———, external, operation for 212
Angina pectoris, cases of 21
——— trachealis, cases and treatment of 458
———, list of writers on 467
Ankle, cases of wounds of the 387
Anomalous disease, case of 168
——— symptoms, curious train of 419
Antshar, description and properties of 257
———, experiments with 260
Apoplexy produced by the use of opium 17
———, case and dissection of 413
———, case of recovery from 485
Apothecaries' act, statement of circumstances connected with the 499
*Arbutus uva ursi*, on the medical properties of 126
Arm, cases of wounds of the 388
Armstrong's, Dr. practical illustrations of typhus 32, 110
Arsenic, ineffi iency of the common tests to detect 529
Artery, carotid, rupture of the 1
———, tied to cure a wound in the face 28
———, tied to facilitate the removal of a tumour 29
Arteries, case of non-pulsation of 455
Asthma alternating with rheumatism 78

B

Balfour's, Dr. letter on rheumatism 288
Barton, Professor, on the *pyrola umbellata* 126
Belgium, report on the military hospitals in 195
Bell's, Mr. surgical observations 140
Black's, Dr. cases of angina pectoris 21
Bladder, cases of wounds of the 382
Bleeding employed to remove debility 80
Blegborough, Mr. on the treatment of croup 476
Blood, increased determination of in *tic douloureux* 130
———, on the transfusion of in hæmorrhage 276
Blood-letting, efficacy of, in dysentery 230
——— in croup 458
Boggie's, Mr. case of fractured tibia 211
Bone, microscopic observations on the structure of 216
BOOKS, NEW, ANNOUNCED.—Dr. Burrows's Commentaries on Mental Derangement, 85. Mr. Copeland Hutchison on Amputation in Gunshot Wounds, 176. An Inquiry into the Effects of Spirituous Liquors on Man, 176. Mr. Hennen on Military Surgery, 265. Translation of Professor Weidman's work on Necrosis, 265. Dr. Burrows's Cursory Remarks on the Bill for regulating Mad Houses, 266, 358. A Medico Chirurgical and Biographical Chart of Medical Science, 358. Dr. A. P. W. Philip's Experimental Inquiry into the Laws of the Vital Functions, 444. Dr. Burrows's Statement of Circumstances connected with the Apothecaries' Act, 444.

### INDEX.

**Books reviewed.**—Medico-Chirurgical Transactions, vol. vii. 18, 125, 207, 291. Dr Armstrong's Practical Illustrations of Typhus, 32, 110. Observations on the Bill for restricting the Practice of Surgery, 46. Dr. Male's Epitome of Juridical or Forensic Medicine, 47. Dr. Griffiths's Essay on the Cause and Prevention of Hepatitis, 139. Mr. Bell's Surgical Observations and Quarterly Report, 140, 305. Dr. Thomson's Report on Military Hospitals in Belgium, 195, 379. Dr. Somers's Medical Suggestions for the Treatment of Dysentery, 224. Dr. Young's Treatise on Consumptive Diseases, 231, 321. Dr. Davis's Inquiry into the Causes of Mortality among Children, 237. Dr. Duncan Stewart on Uterine Hæmorrhage, 331. Medical, Geographical, and Agricultural Report on the Causes of the Epidemic Fever in Coimbatore and other Indian Provinces, in the Years 1809, 10, 11, 392. Dr. Crawford's Experimental Inquiry into the Effects of Tonics, 401. Transactions of the Medical Society of London, 473. Dr. Hutchison's further Observations on the Subject of the proper Period for Amputating in Gun-shot Wounds, 486. Dr. Burrows's Statement of Circumstances connected with the Apothecaries' Act 499

Brain, typhoid inflammation of the 40

——, case of chronic inflammation of the 161

——, case of enlargement of the 504

Brodie, Mr. on the treatment of varicose veins 132

Bronchocele, case of 69

Bubonocele, case of 350

Bulam fever, official report on, 9, 93

C

Cadavery in Sicily, description of 526

Calomel, scruple doses of in dysentery 179

Cancer, inefficacy of compression in 143

Cancerous ulcers, new method of curing 73

Candidates, on the admission of, to the College of Surgeons 81

Cardiac disease, cases of 172, 446

——————, with dropsical effusion 348

Carotid artery, rupture of the 1

Carpo-pedal spasm, case of 448

Catalepsy, an uncommon case of 248

Cerebral inflammation, cases of 262, 355

Chest, description of wounds of the 204

Children, on the causes of mortality amongst 237

*Chordæ tendineæ*, case of rupture of 174

Chorea in an adult, case of 134

———, Dr. Seed's observations on 513

Climate, on the medical effects of 328, 428

Clutterbuck's, Dr. case of diseased action of the heart 482

Coley's, Mr. case of ruptured carotid artery 1

Collier's, Mr. case of wound in the face, requiring ligature of the carotid artery 28

———————— case of aneurism of the femoral artery 125

Congestive typhus, treatment of 122

Connexion between the vascular and nervous systems 513

Consumption, symptoms of 234, 294

——————, morbid appearances in 235

——————, on the causes of 321

——————, on the treatment of 325

—————— cured by *pix liquida* 529

Correspondence, 86, 176, 266, 358, 444, 580

Convulsive diseases, observations on 366

Crampton's, Mr. operation for external aneurism 212

Crawford, Dr. on the effects of tonics 401

Croup, cases of 458

———, on the treatment of 476

D

Davis, Dr. on the causes of mortality among children 237

Debility removed by bleeding 80

Decline, symptoms of 233

Deglutition of air, report of a treatise on 405

Dewar, Dr. on the treatment of sinous ulcers 291

Diabetes, fatal case of 256

Dickson's, Dr. observations on tetanus 219

Diaphragm, cases of wounds of the 379

Digestive organs, affection of the, producing cerebral inflammation 262

*Dura mater*, case of hernia of the 217

Dysentery, use of calomel in 179

—————, medical suggestions for the treatment of 224

Dyspnœa, fatal case of 483

E

Earle, Mr. on the influence of the nervous system in regulating animal heat 129

———— on contractions of the skin after ulcerations 217

———— on hernia of the dura mater 217

Edinburgh, regulations concerning medical degrees in the university of 524
Elaterium given in strangulated hernia 484
Elbow-joint, cases of wounds of 388
Elephantiasis, case of 361
Emetics, on the use of in consumption 326
Emphysema, case of from fractured rib 309
Entero-mesenteric fever, observations on 352
Epidemic, Indian, report on 392
Epilepsy cured by scirrhous affection of the testicle 251
———, pathology and treatment of 473
——— cured by the trephine 528
Erysipelas, infantile, description and treatment of 120
Extra-uterine fœtus, case of 218
——————— gestation, case of 415

F

Face, wounds of the, remarks on 202
Fairbairn's, Mr. case of hydrocephalus 452
Fallopian tube, fœtus contained in 218
Fever, yellow, official report on 9, 92, 182
——, intermittent and remittent, treatment of 226
Flemings, Mr. case of ligature on the carotid artery 2
Fœtus, monstrous, case of 135
Foot, cases of wounds of the 387
Fore arm, cases of wounds of the 389
Forensic medicine, epitome of 47
Fracture, ununited, of the humerus successfully treated 27

G

Gaitskell, Mr. on the treatment of croup 477
—————— case of *lusus naturæ* 481
Gases, intestinal, in the human subject 159
Genital organs, female, *lusus naturæ* of 481
Glasgow, memorial of the university of 519
Glottis, case of an ulcer of the 146
Goodlad's, Mr. case of tumour in the face and neck 28
Gout, inflammatory, terminating in suppuration 77
——, observations on 269
Griffith, Dr. on the cause and prevention of hepatitis 139
Gun-shot wounds, on the proper period for amputating in 486
Guthrie's, Mr. case of wounded peroneal artery 210

H

Hæmato-scrophulous tumour, case of 376
Hæmorrhage, spontaneous, asthma cured by 78
————— in wounds, remarks on 198
—————, uterine, on the treatment of 334
Hæmorrhoids, observations on 449
Hand, cases of wounds of the 389
Head, wounds of the, observations on 199
——, case of fatal injury of the 345
——, case of injury of the 418
Heart, inflammation of the muscular structure of the 208
——, case of diseased action of the 482
——, experiments on the action of the 494
Heat, animal, regulated by the nervous system 129
Hectic fever, symptoms of 232
*Hemetine* procured in a pure state from ipecacuanha 530
Hepatitis, on the cause and prevention of 139
Hernia, cases of, illustrated by dissection 177, 244
——— of the dura mater complicated with hydrocephalus, case of 217
———, femoral, cases of 313
———, strangulated, case of 414
Hernial sac, memoir on the inflammation of 163, 244, 407
Hey, Mr. on the effects of the venereal disease upon the fœtus in utero 299
Hill, Mr. C. N. on contractions of the skin 378
Hip-joint, cases of wounds of the 383
Horsfield's, Dr. experiments with Java poisons 260
Hospitals, military, in Belgium, report on 195
Hottentot Venus, dissection of the 85
——————, on the structure of the genital organs of the 127
Howship, Mr. on the structure of bone 216
———'s, Mr. case of hernia 177
Humerus, removal of the head of the 128
Hutchison, Dr. on amputating in gun-shot wounds 486
Hydatids voided by the urethra 84
Hydrencephalus, observations on 273
Hydrocephalus acutus, case of 452

I

India, report on the epidemic fever of 392
Inflammation, chronic, of the brain, case of 161
————— of the hernial sac, memoir on 163, 244

Inflammation, cerebral, cases of 262, 355
Inspiration, first, of the new born child, memoir on 63, 155, 241
Instruments, new, for diseases of the eye an ear 84
Intermittent fever, treatment of 226
Intestinal gases of the human subject 159
Intestines, cases of wounds of the 381
Issues, on the use of, in consumption 326

J

Johnson's, Dr. case of apoplexy from opium 17
———, Dr. on syncope angens 101, 187
——— case of hæmato-scrophulous tumour 376
Jones's, Dr. analysis of the Spa waters 19
Juridical medicine, epitome of 47
Jurisprudence, medical, remarks on 74, 341, 508

K

Kidnies, analogy between the, and testicles 175
Knee-joint, cases of wounds of the 385

L

Langstaff's, Mr. case of extra-uterine fœtus 218
Lara and Johnson, Drs. case of non-pulsation in the arteries 455
Laryngotomy, method of performing 148
Larynx, case of ossification of the 127
———, on the diseases and wounds of the 144
———, case of disease of the 147
Leacock, Dr. on the transfusion of blood 276
Lectures announced. — Drs. Latham and Southey, on the Practice of Physic, 86. — Mr. Taunton, on Anatomy, 86, 444.—Dr. Clough, on Midwifery, 86. — Mr. Stevenson, on the Eye and Ear, 175.—Dr. Merriman and Dr. Ley on Midwifery, 176, 358.—At the Royal College of Surgeons, 443. — Dr. Clutterbuck, on Medicine and Chemistry, 444. — Mr. Thomson, on Botany, 580.
Lee's, Dr. case of elephantiasis 361
Leg, cases of wounds of the 386
Leper, picture of in Sicily 526
Lepra Arabum, case of 361
Lespagnol's, M. cases of cerebral inflammation 262, 355
Lettsom's, Dr. case of *lumbricus* perforating the intestines 481
——— case of fatal dyspnœa 483
Liquors, spirituous, on the effects of 401
Liver, phosphate of lime contained in a cyst in the 263
———, cases of wounds of the 380
Loins, cases of wounds of the 383
*Lumbricus*, perforating the intestinal canal 481
Lungs, case of abscess of the 254

M

M'Arthur's, Dr. cases of tetanus 221
Male's, Dr. epitome of forensic medicine 47
Marcet, Dr. on the medical properties of stramonium 302
Matéos, Dr. on the degraded state of medicine in Spain 422
*Maunoir's*, M. cafe of monftrous fœtus 135
———, M. on the artificial pupil 207
Medical Miscellanies 76, 170, 252, 345, 417, 513
——— Society of London, anniversary meeting of 358
——— suggestions for the treatment of dysentery 224
——— Society of London, transactions of 473
——— degrees, regulations concerning, in Edinburgh 524
——— benefit society, officers of 580
Medicina Miscellanea 494
——— Laica 526
Medicine, on the degraded state of in Spain 422
Medico-Chirurgical Transactions, review of 18, 207
Memorial of the university of Glasgow 519
Mercury, efficacy of, in congestive typhus 175
———, observations on 450
Meteorological Register for Harwich 88, 267, 268, 360, 581, 582
Milk, best mode of preserving 264
Moisture in houses, on the ill effects of 485
Morel's, Mr. case of removal of the head of the humerus 128
Morrison's, Mr. case of fractured skull 284
Mortality, London bill of 87
Mortimer's, Mr. report on the yellow fever 9, 93, 182
Moulson's, Dr. observations on spasmodic and convulsive diseases 366
Moxa successfully employed in neuralgia 72
Murder, account of a trial for 435
Muscular fibres, on the laceration of 136
Mushrooms, poisoning by, method of curing 264

N

Neck, remarks on wounds of the 204
Negroes eating earth, observations on 500
Nervous system, influence of the, in regulating animal heat 129

Neuralgia of the spermatic cord cured by moxa 72
New-born child, on the first inspiration of the 63, 155, 241
Nicholl's, Dr. case of imperfection of the vagina 224
Nitrate of silver, internally, changes the colour of the skin 137
Norman's, Mr. observations on the trial of Mr.' Donnall 517

O

Obituary 86, 266, 358, 444
Observer, the 269, 445
Odier, Dr. on the last illness of De. Saussure 133
Œsophagus, stricture of the, cured by caustic 150
Oneirodynia, remarkable case of 89
Oopas, or poison tree of Java, description of the 257
Operation for the removal of a large tumour in the face and neck 30
Opium, apoplexy produced by the use of 17
———, on the use of, in uterine hæmorrhage 338
Oration before the London Medical Society, account of the 431
Ovary transformed into a large scirrhous mass 71

P

Palmer's, Dr. cases of croup 458
Parturition, series of misfortunes in 78
Patella, cases of fracture of the 338
Pelvis, cases of wounds of the 383
Penis, cases of wounds of the 383
Percussion, on the efficacy of, in gout 270
Peroneal artery, case of wound of the 210
Pharynx, scirrhous, contraction of the 148
———, ossification of the 127
Philip, Dr. on pulmonary consumption 294
———'s, Dr. experiments on the action of the heart 494
Phosphate of lime contained in the liver 263
Phthisis, dyspeptic, nature and treatment of 296
*Pix liquida* useful in consumption 529
Plagiarism, charge of 171
Plague, on the different kinds of 527
Polydore's, Dr. case of oneirodynia 89
*Porte-feuille*, the 76, 167, 252, 345, 417
Power's, Mr. case of dysentery 170
Pudendum, fatal affection of the 25
———, case of ulceration of the 349
Pupil, artificial, operation for 207
Purgatives, efficacy of in fevers 118
*Pyrola umbellata*, on the medical properties of 126

Q

Quarrier's, Dr. cases of amputation 492

R

Refrigeration employed in the endemia of tropical climates 468
Regulations for medical degrees in Edinburgh 524
Report on the military hospitals in Belgium 195, 379
Rheumatism alternating with asthma 78
———, oriental method of curing 109
———, on the use of percussion in 288
Ribs rendered carious by an abscess of the lungs 254
———, dislocation of, from their cartilages 312
———, fractured, fatal case of 312
Rickets, on the condition of the bones in 216
Rum, on refrigeration by means of, in tropical fevers 468

S

*Saussure De*, on the last illness of 133
Scrotum, case of sloughing of the 152
Seeds's, Dr. observations on chorea 513
Selections, 63, 155, 241, 338, 405, 500
Sepulture, Turkish mode of 526
Seton employed in an ununited fracture of the humerus 27
——— useful in promoting union of the bone 212
Shoulder, cases of wounds of the 387
Sickness, periodical, cases of 479
Silver, nitrate of, changes the colour of the skin 137
Skin, colour of the, changed by internal use of nitrate of silver 137
———, on the contractions of, succeeding ulcerations 217
Skull, fractured, case of 284
Somers, Dr. on the treatment of dysentery 224
Somerville on the structure of the female organs of the Hottentots 127
Spa, analysis of the mineral waters of 19
Spain, on the degraded state of medicine in 422
Spasmodic diseases, observations on 336
Spine, cases of fracture of the 305
Spleen, diseased, case of 372
Spurzheim's, Dr. dissection of the brain, remarks on 425
Stanley, Mr. on inflammation of the heart 208
Stansfield's, Mr. case of ununited fracture of the humerus 27
Stewart, Dr. Duncan, on uterine hæmorrhage 331
Stomach, cases of wounds of the 381
Strammonium, on the medical properties of 302
Surgery, observations on the bill for restricting the practice of 46

Symptoms, anomalous, curious train of 419
Syncope angens, observations on, 101, 187
———, case of 103
———, on the prevention and cure of 191
Syphiloid ulcer cured by *tinctura lyttæ* 417

T

Testes, cases of wounds of the 383
Testicles, scirrhous, epilepsy cured by 251
Testicles, analogy between the, and kidnies 175
Tetanus, observations on 219
———, cases of 221
———, a new remedy for 352
Thetford, account of diseases prevalent at 265
Thigh, case of extraordinary disease of the 76
———, dissection of the 252
———, cases of wounds of the 384
Thomson, Dr. on the military hospitals in Belgium 165
Tibia, fractured, case of 211
*Tinctura lyttæ*, application of, to a syphiloid ulcer 417
Tonics, on the effects of 401
Transactions of the medical society of London 473
Transfusion of blood, experiments on 278
Travers's, Dr. case of ossification of the pharynx 127
Trephine successfully employed in epilepsy 528
Trial for murder, an account of a 435
Tshettick, description and properties of 258
———, experiments with 262
Tumour, hæmato-scrofulous, case of 376
Turkish Bedlam at Constantinople 526
Typhus, practical illustrations of 32, 110
Typhus divided into three varieties 35
———, inflammatory, treatment of 114

U

Ulcer, syphiloid, cured by *tinctura lyttæ* 417
Ulcers, cancerous, new method of curing 73
Ulcers, sinous, on the treatment of 291
University of Glasgow, memorial of 519
——— of Edinburgh, regulations concerning medical degrees in 524
Urethra, hydatids voided by the 84
———, bursting of 152
Urinary concretion, case of 5
Uterine hæmorrhage, on the practice employed in 332
Uterus, case of successful puncture of the 506

V

Vaccination, success of, in Java 170
Vaccine pustule, progress of, arrested by cold water 256
Vagina, case of imperfection of the 224
Varicose veins, on the treatment of 132
Variolous matter, experiments with, on the teats of cows 421
Venereal disease, the effects of on the fœtus in utero 299
Venus, Hottentot, dissection of 85

W

Ward's, Mr. case of urinary concretion 5
Wardrop, Mr. on the laceration of muscular fibres 136
Wood's, Mr. account of a fatal affection of the pudendum 25
——— case of chorea 134
——— case of Cæsarian operation 135
Woodforde's, Dr. case of strangulated hernia 484
——— case of apoplexy 485
Wound, its relation with juridical medicine 74
Wounds of the head, remarks on 199
——— face and neck, remarks on 202
——— chest, description of 204

Y

Yeats's, Dr. case of diseased spleen 372
Yellow fever, Mr. Mortimer's report on 9, 93, 182
———, cases of second attack of 99
Young, Dr. on consumptive diseases 231, 321

References to the Plates.

Mr. Howship's Case of Hernia . . . . . . . . . . . . . 177
Dr. Lee's Case of Elephantiasis . . . . . . . . . . . 361

PRINTED BY W. THORNE, RED LION COURT, FLEET STREET.

